# L-carnitine ameliorated fasting-induced fatigue, hunger, and metabolic abnormalities in patients with metabolic syndrome: a randomized controlled study

**DOI:** 10.1186/1475-2891-13-110

**Published:** 2014-11-26

**Authors:** Jun-jie Zhang, Zhi-bing Wu, You-jin Cai, Bin Ke, Ying-juan Huang, Chao-ping Qiu, Yu-bing Yang, Lan-ying Shi, Jian Qin

**Affiliations:** Department of Traditional Chinese Medicine, First Affiliated Hospital of Sun Yat-Sen University, No. 58 Zhongshan 2nd Road, Guangzhou, 510080 China; Seasonal Febrile Diseases Section of First Affiliated Hospital of Guangzhou University of Chinese Medicine, Guangzhou, China

**Keywords:** Metabolic syndrome, Very-low-calorie-diet, L-carnitine, Weight loss

## Abstract

**Background:**

The present study aimed to determine that whether L-carnitine infusion could ameliorate fasting-induced adverse effects and improve outcomes.

**Method:**

In this 7-day, randomized, single-blind, placebo-controlled, pilot study, 15 metabolic syndrome (MetS) patients (11/4 F/M; age 46.9 ± 9.14 years; body mass index [BMI] 28.2 ± 1.8 kg/m^2^) were in the L-carnitine group (LC) and 15 (10/5 F/M; age 46.8 ± 10.9 years; BMI 27.1 ± 2.3 kg/m^2^) were in the control group (CT). All participants underwent a 5-day modified fasting therapy introduced with 2-day moderate calorie restriction. Patients in the LC group received 4 g/day of intravenous L-carnitine, while patients in the CT group were injected with saline. Blood pressure (BP), anthropometric characteristics, markers of liver function, metabolic indices (plasma glucose, lipid profiles, uric acid, free fatty acid and insulin) and hypersensitivity C-reactive protein were measured. Perceived hunger was recorded daily by self-rating visual analogue scales. Fatigue was evaluated by Wessely and Powell scores.

**Results:**

In contrast to the CT group, total cholesterol, alanine aminotransferase, systolic and diastolic BP did not change significantly in the LC group after prolonged fasting. There were significant differences in weight loss (LC −4.6 ± 0.9 vs. CT −3.2 ± 1.1 kg, *P* = 0.03), and waist circumference (LC −5.0 ± 2.2 vs. CT −1.7 ± 1.16 cm, *P* < 0.001), waist hip ratio (LC −0.023 ± 0.017 vs. CT 0.012 ± 0.01, *P* < 0.001), insulin concentration (LC −9.9 ± 3.58 vs. CT −6.32 ± 3.44 µU/mL, *P* = 0.046), and γ-glutamyltransferase concentration (LC −7.07 ± 6.82 vs. CT −2.07 ± 4.18, *P* = 0.024). Perceived hunger scores were significantly increased (P < 0.05) in the CT group during starvation, which was alleviated with L-carnitine administration in the LC group. Physical fatigue (LC −3.2 ± 3.17 vs. CT 1.8 ± 2.04, *P* < 0.001) and fatigue severity (LC −11.6 ± 8.38 vs. CT 8.18 ± 7.32, *P* < 0.001) were significantly reduced in the LC group but were aggravated in the CT group.

**Conclusion:**

Intravenous L-carnitine can ameliorate fasting-induced hunger, fatigue, cholesterol abnormalities and hepatic metabolic changes and facilitate fasting-induced weight loss in MetS patients.

**Trial registration:**

ChiCTR-TNRC-12002835.

## Introduction

Metabolic syndrome (MetS) is defined as a cluster of cardiovascular risk factors (i.e., abdominal obesity, hyperinsulinaemia, atherogenic dyslipidemia, hypertension, hypercogauability) that increase the risk of developing co-existing coronary heart disease (CHD) and type 2 diabetes [1]. In recent years, the incidence of chronic diseases associated with MetS, such as hypertension, diabetes and dyslipidemia, have rapidly increased in China [2]. In 2009, modified fasting therapy was initially applied as a therapeutic option for the management of overweight, obesity and MetS in South China [3].

Modified fasting therapy is defined as a form of very-low-calorie-diet (VLCDs), which has been applied in an integrative medicine ward in Germany for decades [4]. It is a regimen whereby juicy food intake is limited to 350 Kcal per day in the short-term. As an effective approach to MetS, modified fasting therapy has long been employed as a lifestyle modification in Europe. However, when it was initially applied to the Chinese population, fasting-induced persistence fatigue and intense hunger were found to be the main obstacles to achieving therapeutic benefits [3]. Energy deficiency may be the leading cause of fatigue and intense hunger during the fasting period. It is well known that lipid mobilization is the main metabolic adaptation to starvation. Accumulating evidence suggested that facilitating fatty acid oxidation with free L-carnitine (β-hydroxy-γ-N-trimethylaminobutyric acid) contributes to energy production [5].

L-Carnitine is an essential nutrient in lipid metabolism, which promotes the transportation of long-chain-fatty-acids across the mitochondrial membrane, thus allowing the cells to break down fat and release energy from reserves of stored fat [6, 7]. All tissues that use fatty acids as an energy source require carnitine for normal function during fatty acid oxidation. In addition to its requisite role in fatty acid oxidation, it can facilitate glucose disposal by modifying the mitochondrial acetyl-CoA/CoA ratio [8]. Excessive acetyl-CoA production in the process of β-oxidation inhibits the tricarboxylic acid (TCA) cycle, leading to mitochondria stress in hepatocytes [9, 10]. Accordingly, Calabrese et al. proposed that free L-carnitine might contribute to the alleviation of starvation-related stress [11]. In addition, evidence strongly demonstrates that long-term oral L-carnitine administration is effective for fatigue during weight loss induced by chronic disease [12]. Therefore, we postulated that L-carnitine supplementation could ameliorate the adverse effects of starvation.

However, when compared to traditional orally administrated drugs, the pharmacokinetic properties of L-carnitine are complex, since it exhibits poor intestinal absorption and bioavailability, very high renal clearance, and uptake into tissues through a high affinity transporter [13]. Therefore, repeated-dose carnitine supplements only modestly increase the plasma L-carnitine concentration in muscular tissue even when very high oral doses are administrated, (i.e., more than 2 g per day) [14]. L-carnitine administrated intravenously should substantially increase the L-carnitine concentration. However, since the tubular reabsorption occurs via an active transporter, once the transporter is saturated by the high plasma L-carnitine concentrations achieved by intravenous administration, the circulating L-carnitine is rapidly excreted. In addition, because the acylcarnitine ester pool in the extracellular fluid is increased following the L-carnitine infusion, L-carnitine can enter the intracellular pool for utilization before clearance [15]. Accordingly, we proposed that a large dose of L-carnitine administrated intravenously was responsible for alleviating starvation discomfort including persistence fatigue and intense hunger over the short term.

Recently, Awad et al. also posed the viewpoint that L-carnitine should help attenuate the adverse effects of perioperative fasting and lead to improved patient outcomes [16]. To verify this hypothesis, we designed a randomized controlled study to determine the effect of 4 g/day of intravenous L-carnitine administration on fatigue, hunger, body mass, lipid profile, and other CHD risk factors in MetS patients during a modified fasting period.

## Subjects and methods

### Subjects

This randomized, single-blinded, placebo-controlled clinical study was conducted in MetS patients at the First Affiliated Hospital of Sun Yat-Sen University in Guang Zhou, China and has been registered with the Chinese Clinical Trial Registry (No. *ChiCTR-TNRC-12002835*). 30 overweight or obese (body mass index [BMI] ≧25.0 kg/m^2^) patients aged from 27–65 years in whom MetS was diagnosed according to the updated National Cholesterol Education Program/Adult treatment Panel III criteria for Asians were enrolled into the study. The subjects had to meet at least 3 of the following components: waist circumference ≧80 cm in women and ≧90 cm in men; triglycerides (TG) ≥1.7 mmol/L; high-density lipoprotein (HDL) cholesterol <1.03 mmol/L for men or <1.29 mmol/L for women; blood pressure ≥130/85 mmHg or current use of antihypertensive medications; or fasting glucose ≥5.6 mmol/L [17].

The exclusion criteria were: females who were pregnant or experiencing a menstrual period, type 2 diabetes treated using an insulin regimen, malignancy, hematopathy, active tuberculosis, peptic ulcer with gastric bleeding, binge-eating disorder, psychiatric disease, congestive heart failure, clinically relevant renal or hepatic insufficiency, unstable coronary artery disease, cancer or a history of cancer. In addition, patients were not included in the study if they were on a carnitine supplement or on other drugs known to influence lipid metabolism, such as β-blockers, glucocorticoids, or lipid-lowering agents during the previous two weeks. Baseline characteristics of patients are presented in Table 1.

**Table 1:** **Demographic characteristics of MetS patients in the two treatment groups** ^**a**^

	Control group (Fasting alone)	Carnitine group (Fasting + L-carnitine)	***P***-value^b^
Number	15	15	1.00
Gender (Female/Male)	10/5	11/4	0.702
Age (year)	46.8 ± 10.9(27–65)	46.9 ± 9.14(37–60)	0.973
Body mass (kg)	74.2 ± 6.9(59–86)	75.1 ± 7.5(64.1-93)	0.721
BMI (kg/m^2^)	27.4 ± 2.0(25–32.6)	28.2 ± 1.8(26.1-32.4)	0.348
WC (cm)	90.4 ± 4.99(83–98)	94.1 ± 6.0(84–107)	0.229
Metabolic disease			
Hypertension	4	5	0.702
Impaired glucose tolerance	3	5	0.426
Dyslipidemia	12	13	0.638

*Abbreviation:* *BMI* body mass index, *WC* waist circumference.

^a^Variables are expressed as mean ± SD.

^b^ *P*-value was determined by unpaired test.

### Randomization and blinding

**Figure 1:**
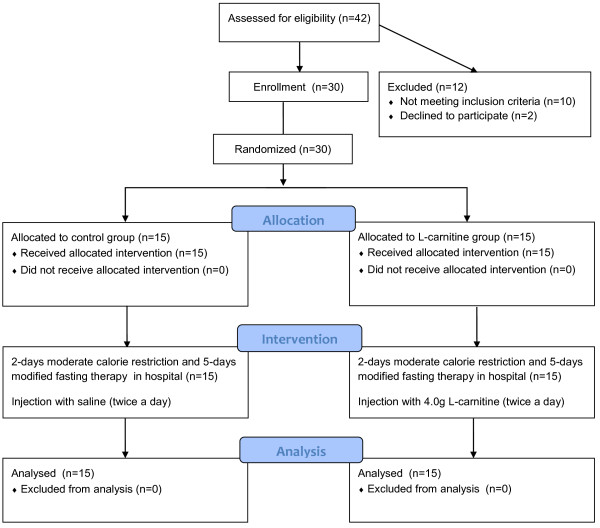
**Flow diagram.** Study participant eligibility assessment, enrollment, group allocation and analysis. Subjects recruited for the study were assessed and those eligible were enrolled. Participants were randomized into L-carnitine and control groups. Patients were blinded from groups. Statistical analysis was performed on data from all subjects completing the study.

The study was conducted between October and December 2012. Subjects were stratified by gender to achieve balance of baseline characteristics. Eligible MetS patients were randomly allocated into either the L-carnitine (LC) group or the Control (CT) group based on a random number table, generated by SPSS 13.0 software (Figure 1). Allocation was determined by investigator not involved in the assessment or intervention. Patients were blinded to intervention during the period. Physicians who performed baseline and outcome assessments were also blinded to the randomization status.

### Intervention

Patients in the LC group received 4 g/d L-carnitine infusion (2 g, twice a day) (Lanling Pharmaceutical CO., LTD, Changzhou, China) from the beginning of the pre-fasting day at 9 am and 4 pm to the last day of fasting. 2 g L-carnitine was dissolved into 20 mL saline for intravenous injection in patients in the LC group. Subjects in the control group were injected with 30 mL saline twice daily as placebo control. There was no difference between two types of colorless transparent injection from appearance administered to both groups. In cases where severe adverse events occurred that were considered to be related to L-carnitine, including severe gastrointestinal upset and seizure, the participant was excluded from the study.

### Diet and exercise

Participants were encouraged to maintain their normal dietary intake during an initial baseline week. The fasting treatment consisted of 2 days with moderate calorie restriction (700–800 Kcal/day), 5 days of intense calorie restriction (200 Kcal/day), followed by 3 days with the stepwise reintroduction of a normal diet. Fasting was initiated with the intake of 10–20 g of thenardite powder on fasting day 1 for bowel-cleansing purposes. During the 5-day fasting period, the subjects were asked to drink at least 2 L of water. During the fasting period, they were prescribed a diet of 200 Kcal/d that consist of one serving of the liquid meal replacement and two cups of light vegetable soup. Each serving of the liquid diet provided approximately 150 Kcal: 7.9 g protein, 2.8 g fat, and 22 g carbohydrate. The liquid diet was prepared by mixing the powdered product with 250 mL water and was to be consumed at dinner. Two bowls of vegetable soup provided about 50 Kcal and were given at breakfast and lunch. All patients were required to engage in low-level physical activity consisting of 2 hours slow walking per day. Furthermore, patients were recommended to avoid alcohol, coffee and tea during the study. All participants were compulsory hospitalized during 2-days pre-fasting and during 5-days fasting period.

### Primary and secondary outcomes

The primary endpoint was defined as significant (P < 0.05) changes in weight loss. A power analysis (α = 0.05 and power = 0.90), based on data where a difference would be significant if the weight loss were 1 kg more than that in CT group with an expected standard deviation of 0.75, indicated that 13 patients would be needed in each group to detect significant difference [18]. A predefined drop-out level was estimated to be about 15%.

The secondary endpoint included changes in glucose, insulin, HOMA-IR, blood pressure, lipid profiles, uric acid, free fatty acids, hepatic profile, and the sensation of fatigue and hunger.

### Clinical evaluation

Body mass was measured daily at 8 am on a balance-beam scale with subjects wearing light clothing and no shoes. Blood pressure measurements were also performed daily at 8 am using a manual sphygmomanometer. Two measurements were taken in a sitting position, after 10 minutes rest. The measurement was performed using the cuff enclosing rubber bladder, 12 cm wide and 35 cm long. The mean blood pressure value was used for statistical analysis. Waist circumference and hip circumference were measured according to the procedures of Calloway et al. prior to and after fasting [19]. In addition, patients rated throughout day 0 and day 7 their perceived hunger on a 10-point Likert scale ranging from not at all (0) to very much (10). This approach to assessment of hunger has been described and evaluated previously [20]. Fatigue was evaluated by Wessely and Powell scores consisting of physical fatigue, mental fatigue, and fatigue severity [21].

### Biochemical evaluations

Venous blood samples were drawn at 7 am after 12-hour overnight fasting from an antecubital vein at day 0 and day 7 of the study. Plasma glucose concentration was determined by an enzymatic colorimetric test. Radioimmunoassay was used for measurement of insulin (Diagnostic Products, Los Angeles, CA). Free fatty acid (FFA) levels were determined enzymatically using a NEFA test kit (Sekisui Medical Co., LTD, Tokyo, Japan). Homeostasis model assessment was used to measure insulin resistance (HOMA-IR) using the following equations: HOMA-IR = [FPG(mmol/L) × fasting insulin (µU/mL)]/22.5.

Total cholesterol (TC) and triglycerides (TG) were assayed using the enzymatic colorimetric test (Human, Wiesbaden, Germany) with lipid clearing factor. High-density lipoprotein (HDL) cholesterol and low-density lipoprotein (LDL) cholesterol were measured enzymatically by direct method. Immunoturbidimetric assays were used to determine serum apolipoprotein A1 (apoA1), apolipoprotein B (apoB), lipoprotein (a) (Lp (a)) [Shanghai Kehua Bio-engineering Co., LTD, Shanghai, China] and hypersensitivity C reactive protein (Hs-CRP) [Orion Diagnostica Oy, Espoo, Finland] levels. Colorimetric/fluorometric assay kits [Biovision Incorporated, Milpitas, USA] were used to measure aspartate aminotransferase (AST), alanine aminotransferase (ALT) and γ-glutamyltransferase (γ-GGT). All assays were performed in the central laboratory of the First Affiliated Hospital of Sun Yat-Sen University.

### Ethical consideration

Written informed consent was obtained from all participants before enrollment. The study design was approved by the Ethics Committee of the first Affiliated Hospital of Sun Yat-Sen University ([2012]248). We obeyed the principles of the 1964 Declaration of Helsinki and its later amendments [22].

### Statistical analysis

Results are given as mean ± standard deviation or standard error of the mean, as indicated. Paired and independent-sample *t* tests were used for comparing the baseline data, comparison of data at baseline and post-fasting, and the changes in variables between groups. With-in group variations for body mass change were tested using a repeated measures analysis of variance. If there was a main effect, the Bonferroni correction was used. Differences were regarded as significant at *p* <0.05. Statistical analyses were performed with SPSS version 13.0 software (SPSS, Inc., Chicago, IL).

## Results

### Change in anthropometric measurements

The baseline characteristics of MetS patients are shown in Table 1. There was no significant difference in age and body mass between groups. Mean body mass, BMI, waist circumference, hip circumference, systolic and diastolic blood pressure at baseline and post-fasting are presented in Table 2. There was a significant decrease in these parameters during fasting in the CT group. Blood pressure did not change significantly during starvation in the LC group. Waist hip ratio (WHR) increased significantly after semi-starvation in the CT group (baseline vs. post-fasting, *P* < 0.001), while it decreased significantly in the LC group (baseline vs. post-fasting, *P* < 0.001).

**Table 2:** **Anthropometric measurement in MetS patients at baseline and post-fasting in two groups** ^**a**^

	Control group				Carnitine group				^Δ^ ***P***-value
	Baseline	Post-fasting	***P***-value^b^	Changes	Baseline	Post-fasting	***P***-value^b^	Changes	
Body mass (kg)	74.2 ± 6.9	71 ± 6.6	<0.001	-3.2 ± 1.1	75.1 ± 8.0	70.5 ± 7.7	<0.001	-4.6 ± 0.9	0.04
BMI (kg/m^2^)	27.4 ± 2.0	26.2 ± 2.1	<0.001	-1.02 ± 0.28	28.2 ± 1.8	26.5 ± 1.8	<0.001	-1.6 ± 0.39	<0.001
WC (cm)	90.6 ± 8.95	88.9 ± 8.6	<0.001	-1.7 ± 1.16	94.1 ± 6.0	89.1 ± 6.1	<0.001	-5.0 ± 2.2	<0.001
HC (cm)	102.5 ± 4.66	99.8 ± 6.78	<0.001	-2.7 ± 1.57	103.8 ± 3.96	100.9 ± 4.11	<0.001	-2.9 ± 2.03	0.78
WHR	0.88 ± 0.05	0.89 ± 0.08	<0.001	0.012 ± 0.01	0.91 ± 0.07	0.88 ± 0.06	<0.001	-0.023 ± 0.017	<0.001
SBP (mmHg)	135.7 ± 15.6	126.4 ± 8.9	<0.001	-9.26 ± 7.83	120.6 ± 15.45	115.2 ± 8.89	0.190	-5.35 ± 14.48	0.064
DBP (mmHg)	80.8 ± 8.4	76.1 ± 6.0	0.001	-3.73 ± 3.41	78.9 ± 9.4	75.6 ± 4.73	0.202	-3.29 ± 9.15	0.649

*Abbreviation:* *BMI* body mass index, *DBP* diastolic blood pressure, *HC* hip circumference, *SBP* systolic blood pressure, *WC* waist circumference, *WHR* waist hip ratio.

^a^Variables are expressed as mean ± SD.

^b^ *P*-value was determined by paired test between baseline and post-fasting.

^**Δ**^ *P*-value was determined by unpaired test between groups in difference.

**Figure 2:**
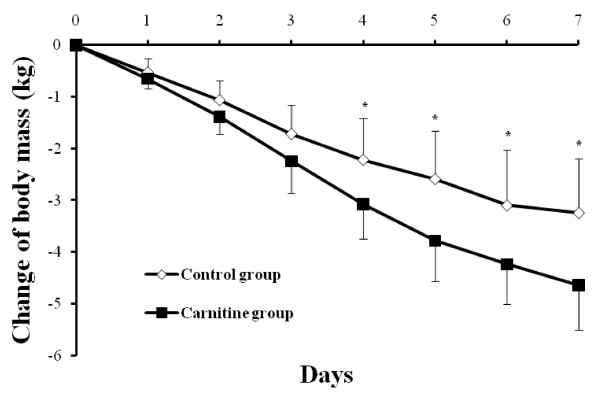
**Changes in body mass during pre-fasting and fasting period.** Variables were presented as mean ± SD. ^*^ *P* -value <0.05, carnitine vs. control group.

As shown in Figure 2, body mass declined during fasting in the LC group to a significantly larger degree than that observed in the CT group (LC vs. CT, P = 0.04). As presented in Table 2, waist circumference reduction in the LC group was greater than in the CT group (LC vs. CT, P < 0.001). There was no significant difference in the reduction of hip circumference between both groups (LC vs. CT, *P* = 0.78). However, WHR decreased in the LC group, while it increased in the CT group after fasting (LC vs. CT, *P* < 0.001). Changes in systolic and diastolic blood pressure showed no significant difference between the LC and CT groups.

### Change in lipid profiles

TC and LDL cholesterol concentrations in the CT group were significantly increased, while TC concentration in the LC group changed without any significant difference after short-term fasting as shown in Table 3. The change in TC concentration in the CT group was markedly larger than that in the LC group (CT vs. LC, *P* = 0.038). There were significant reductions in HDL cholesterol and TG concentration after weight loss in both groups but no statistical difference in these variables after fasting between the two groups.

**Table 3:** **Lipid profiles in MetS patients at baseline and post-fasting in two groups** ^**a**^

	Control group				Carnitine group				^Δ^P-value
	Baseline	Post-fasting	P-value^b^	Changes	Baseline	Post-fasting	P-value^b^	Changes	
TC (mmol/L)	5.34 ± 1.14	5.9 ± 1.28	0.004	0.60 ± 0.68	5.38 ± 0.92	5.43 ± 0.66	0.5	0.1 ± 0.52	0.038
TG (mmol/L)	1.69 ± 0.69	1.11 ± 0.39	0.001	-0.58 ± 0.54	1.72 ± 0.77	0.97 ± 0.22	0.006	-0.60 ± 0.65	0.927
HDL-c (mmol/L)	1.15 ± 0.22	1.02 ± 0.24	0.002	-0.13 ± 0.13	1.11 ± 0.26	1.01 ± 0.19	0.019	-0.11 ± 0.15	0.807
LDL-c (mmol/L)	3.72 ± 1.08	4.57 ± 1.19	0.001	0.86 ± 0.79	3.78 ± 0.96	4.24 ± 0.69	0.018	0.54 ± 0.71	0.279
ApoA1 (mg/L)	1.11 ± 0.24	0.93 ± 0.17	0.007	-0.18 ± 0.22	1.32 ± 0.19	1.16 ± 0.17	0.005	-0.15 ± 0.16	0.745
ApoB (mg/L)	0.94 ± 0.25	1.10 ± 0.22	0.002	0.17 ± 0.17	0.96 ± 0.14	1.12 ± 0.15	<0.001	0.19 ± 0.12	0.645
ApoA1/ApoB	1.14 ± 0.23	0.85 ± 0.18	0.002	-0.29 ± 0.29	1.38 ± 0.25	1.03 ± 0.25	0.005	-0.36 ± 0.25	0.481
Lp(a) (mg/L)	273.4 ± 91.39	422.3 ± 144.83	0.003	148.9 ± 163.7	233.4 ± 90.36	362.8 ± 191.68	0.003	135.1 ± 131.7	0.810

*Abbreviation:* *apoA1* apolipoprotein A1, *apoB* apolipoprotein B, *lp(a)* lipoprotein (a), *HDL-c* High density lipoprotein cholesterol, *LDL-c* Low density lipoprotein cholesterol, *TC* Total cholesterol.

^a^Variables are expressed as mean ± SD.

^b^P-value was determined by paired test.

^**Δ**^ *P*-value was determined by unpaired test between groups in difference.

ApoA1 concentration and the apoA1/apoB ratio in both groups was notably reduced, while Lp (a) and apoB concentrations in both groups increased significantly during the fasting period as presented in Table 3. The changes in these parameters during fasting showed no significant difference in both groups.

### Change in other metabolic substrates and liver enzymes

Serum FFA and uric acid (UA) concentrations increased significantly in both groups after fasting as shown in Table 4. However, the increased values exhibited no statistical difference between the LC group and CT group. Insulin and blood glucose concentrations decreased remarkably during the fasting period as shown in Table 4. Meanwhile, decreased values of FBG during fasting revealed no statistical difference between the LC group and CT group (LC vs. CT, *P* = 0.066). There was a significant reduction in HOMA-IR during fasting as presented in Table 4.

**Table 4:** **Laboratory parameters in MetS patients at baseline and post-fasting in two groups** ^**a**^

	Control group				Carnitine group				^Δ^ ***P***-value
	Baseline	Post-fasting	***P***-value^b^	Change	Baseline	Post-fasting	***P***-value^b^	Change	
FBG (mmol/L)	5.1 ± 0.92	3.8 ± 0.86	0.007	-1.3 ± 0.93	5.2 ± 0.61	4.2 ± 0.66	0.001	-1.07 ± 0.76	0.066
FINS (µU/mL)	16.64 ± 4.98	10.32 ± 5.44	<0.001	-6.32 ± 3.44	17.87 ± 5.81	8.30 ± 4.05	<0.001	-9.9 ± 3.58	0.046
HOMA-IR	3.77 ± 1.35	1.74 ± 0.98	<0.001	-2.03 ± 1.33	4.13 ± 1.38	1.55 ± 0.87	<0.001	-2.78 ± 1.07	0.078
HS-CRP (mg/L)	2.3 ± 3.74	2.2 ± 3.22	0.716	-0.04 ± 0.38	3.06 ± 1.69	2.7 ± 1.45	0.794	-0.09 ± 1.64	0.991
UA (umol/L)	322.2 ± 93.92	527.6 ± 175.1	<0.001	205.4 ± 167	329.3 ± 101.82	460.16 ± 161.33	0.003	130.8 ± 132.9	0.196
FFA (umol/L)	444.4 ± 120.3	990 ± 469.5	0.004	545.6 ± 450.3	454.0 ± 181.51	1187.9 ± 490.96	0.002	831.1 ± 573.9	0.076
AST (U/L)	22.2 ± 5.87	30.6 ± 15.7	0.014	8.39 ± 11.63	26.1 ± 7.74	33.9 ± 17.01	0.045	7.71 ± 12.99	0.883
ALT (U/L)	21.3 ± 11.98	31.3 ± 26.31	0.030	9.99 ± 15.96	25.7 ± 10.76	29.3 ± 14.13	0.112	5.5 ± 12.08	0.403
γ-GGT (U/L)	24.7 ± 10.7	22.3 ± 9.32	0.076	-2.07 ± 4.18	30.2 ± 13.97	22.6 ± 8.62	0.002	-7.07 ± 6.82	0.024

*Abbreviation:* *γ-GGT* γ-glutamyltransferase, *AST* Aspartate aminotransferase, *ALT* Alanine aminotransferase, *FBP* Fasting blood glucose, *FINS* Fasting insulin, *HOMA-IR* Homeostasis model assessment- Insulin Resistance, *Hs-CRP* Hypersensitivity C reaction protein, *FFA* free fatty acid, *UA* uric acid.

^a^Variables are expressed as mean ± SD.

^b^ *P*-value was determined by paired test between baseline and post-fasting.

^**Δ**^ *P*-value was determined by unpaired test between groups in difference.

Furthermore, reductions in insulin concentration were significantly larger in the LC group than in the CT group (LC vs. CT, *P* = 0.046). However, reduction of HOMA-IR between groups was not significantly different (LC vs. CT, *P* = 0.078).

Table 4 also showed that AST and ALT concentrations increased significantly in the CT group, whereas minimal changes in ALT concentration were observed during fasting in the LC group. On the other hand, γ-GGT concentration decreased significantly in the LC group, while there were no statistical changes in the CT group. Furthermore, the decreased value during the fasting period in the LC group was larger than that in the CT group (LC vs. CT, *P* = 0.024). hs-CRP concentration remained stable with no significant changes during fasting in both groups.

### Change in perceived hunger and fatigue

In the CT group, hunger score significantly increased as compared to baseline during the study period. Perceived hunger score at baseline was 4.6 ± 0.8. It gradually and significantly increased over the following days compared to baseline: 4.8 ± 1.11 on day 2 (*P* = 0.486), 5.8 ± 1.05 on day 3 (*P* < 0.001), 6.01 ± 1.12 on day 4 (*P* < 0.001), 6.27 ± 1.81 on day 5 (*P* = 0.003), 6.40 ± 1.96 on day 6 (*P* = 0.005), and 6.20 ± 2.37 on day 7 (*P* = 0.03).

**Figure 3:**
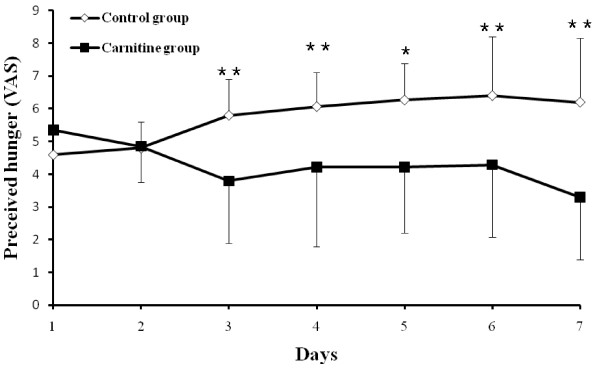
**Perceived hunger scores in groups during the pre-fasting and fasting period.** Variables were presented as mean ± SD. Perceived hunger scores were calculated as the difference between control and carnitine during pre-fasting and fasting period. ^*^ *P* -value <0.05, ^**^ *P* -value <0.01, carnitine vs. control group.

When compared with baseline, perceived hunger score in the LC group decreased. Hunger score at baseline was 4.9 ± 1.11. Subsequently, it declined steadily: 4.6 ± 1.88 on day 2 (*P* = 0.389), 3.57 ± 2.43 on day 3 (*P* = 0.036), 4.14 ± 2.01 on day 4 (*P* = 0.127), 4.07 ± 2.21 on day 5 (*P* = 0.127), 4.14 ± 1.91 on day 6 (*P* = 0.136) and 3.14 ± 1.75 on day 7 (*P* = 0.003). As shown in Figure 3, hunger evidently declined in the LC group when compared to the CT group during the fasting day.

Table 5 showed that in the CT group, mental fatigue did not change significantly, while physical and fatigue severity score significantly increased after modified fasting therapy. However, L-carnitine remarkably reduced physical and mental fatigue and fatigue severity scores during starvation. In addition, as compared to the CT group, L-carnitine improved physical fatigue (LC vs. CT, *P* < 0.001), mental fatigue (LC vs. CT, *P* = 0.001), and fatigue severity (LC vs. CT, *P* < 0.001).

**Table 5:** **Fatigue scores in MetS patients at baseline and post-fasting in two groups** ^**a**^

	Control group				Carnitine group				^Δ^P-value
	Baseline	Post-fasting	***P***-value^b^	Change	Baseline	Post-fasting	***P***-value^b^	Change	
Physical fatigue (0–16)	5.1 ± 2.77	6.9 ± 3.75	0.014	1.8 ± 2.04	6.5 ± 4.11	3.3 ± 3.50	0.001	-3.2 ± 3.17	<0.001
Mental fatigue (0–10)	3.3 ± 2.24	3.3 ± 1.95	1.00	0.0 ± 1.79	3.9 ± 2.25	1.8 ± 1.99	<0.001	-2.2 ± 1.23	0.001
Fatigue Severity (9–63)	27.4 ± 11.26	35.55 ± 11.02	0.004	8.2 ± 7.32	34.8 ± 14.65	23.2 ± 11.72	<0.001	-11.6 ± 8.38	<0.001

^a^Variables are expressed as mean ± SD.

^b^P-value was determined by paired test.

^**Δ**^ *P*-value was determined by unpaired test between groups in difference.

### Tolerability

All the participants went through short-term modified fasting without hypoglycemia or other severe adverse reactions. Furthermore, there were no drop outs during the study period. Of the participants in the CT group, one man experienced mild gingival bleeding and one woman had premature menstruation during the fasting period. As for the LC group, one man experienced mild gingival bleeding and two women complained of transient nausea during the study period.

## Discussion

This is the first study to show that the intravenous administration of 4 g/day of L-carnitine reduced the perception of hunger and fatigue and magnified body mass reduction in MetS patients during modified fasting. We also found that it was able to normalize sitting blood pressure and alleviate cholesterol and hepatic enzyme abnormalities induced by starvation.

In our study, the results showed that L-carnitine administration could alleviate perceived hunger in MetS patients during the modified fasting period. It was well known to us that modified fasting therapy was an effective approach for weight management, however, persistence hunger and fatigue during the fasting period affected compliance with modified fasting therapy. Modified fasting therapy has been used as an effective regime for initial lifestyle modification for chronic metabolic disease, and has been utilized for decades in Germany. However, until now weight reduction induced by modified fasting therapy has not been proved to be effective for long-term weight management. Moreover, it has been proven that gorging induced by obvious hunger after zero-calorie fasting has a harmful effect on health, especially with respect to weight gain [23]. In our study, we demonstrated that perceived hunger scores were significantly increased during the modified fasting period, while intravenously administered high-dose L-carnitine weakened the sensation of hunger during this period. Thus, we hypothesized that hunger-free fasting was more effective than traditional fasting on long-term weight management, although this requires further study.

Furthermore, it has been reported that fatigue induced by progressive disease is often coupled with weight loss and anorexia [24]. However, evidence proved that orally administered L-carnitine was effective in alleviating fatigue syndrome induced by chronic metabolic disease and improving quality of life [25]. In our study, L-carnitine infusion not only ameliorated the sensation of hunger, but improved fatigue with significant weight loss during the modified fasting period. White AM and colleagues previously reported that blood concentrations of ketone bodies were directly associated with fatigue during weight loss under a low carbohydrate/high protein diet [26]. L-carnitine administration was able to reduce the production of ketone bodies in the hepatocyte under starvation [27]. Therefore, it can be postulated that reduction of blood concentrations of ketone bodies with L-carnitine administration contributed to the improvement in fatigue syndrome.

Interestingly, together with fatigue improvement, weight loss and anorexia was significantly promoted with intravenous L-carnitine administration during modified fasting therapy in our study. Furthermore, our results suggest that increased WHR during the period was reversed by L-carnitine supplementation. In addition, a reduction in WC was also seen in the LC group. Based on these results, it was inferred that L-carnitine was able to promote lipolysis and contribute to preservation of lean body mass during modified fasting.

In experimental research, oral L-carnitine supplementation was proved to increase total energy expenditure in obese rats with insulin resistance [28]. In addition, L-carnitine supplementation could promote weight loss in calorie restricted cats that received 40% of maintenance energy requirement [29]. Subsequently, they demonstrated that increasing the basal metabolic rate was the underlying cause of L-carnitine on weight loss [30]. However, these results differ from previous results, which revealed that oral L-carnitine supplementation had no effect on weight loss during calorie restriction in impaired glucose metabolic patients [31] and during aerobic training in obese women [32]. These paradoxical results may be related to the poor bioavailability following oral L-carnitine administration. Oral L-carnitine only mildly increased plasma and muscular L-carnitine concentrations even when very high oral doses are administrated as compared with intravenous administration. Despite high clearance of intravenous administration, L-carnitine could enter the intracellular pool for utilization before clearance during the modified fasting period.

In addition, our study suggested that L-carnitine supplementation facilitated insulin concentration reduction with blood glucose stabilization during modified fasting. It has been shown that hypercarnitinemia stimulated nonoxidative glucose disposal in healthy subjects [28]. Prohibited glucose disposal under starvation contributes to the prevention of hypoglycemia; however, L-carnitine administration induced no hypoglycemia events in our study. On one hand, glucose homeostasis in this situation may be attributed to a significant decline in insulin secretion. Interestingly, blood glucose concentration appeared to decrease with intravenous administration of L-carnitine. Accordingly, lipolysis promotion with L-carnitine increasing endogenous glucose synthesis may be the main cause of glucose homeostasis.

Recently, it has been demonstrated that L-carnitine supplementation can improve insulin resistance and glucose disposal in insulin-resistant humans and rats [33–35]. However, our results showed that it exerted no obvious effect on HOMR-IR improvement in MetS patients during modified fasting, although there was significant insulin concentration reduction. Modified fasting may play a primary role in HOMR-IR reduction, not L-carnitine. Nevertheless, the combination of modified fasting plus L-carnitine could be applied as an effective approach for hyperinsulinemia and insulin resistance. The mechanism underlying this combination may be correlated with carnitine homeostasis and distribution.

The intracellular carnitine homeostasis and distribution are controlled by the novel organic cation transporter (OCTN) among which OCTN2 is the most important. Its main functions are to operate on intestinal and renal reabsorption of L-carnitine and play a vital role in the distribution. It has been demonstrated that fasting and calorie restriction significantly increased the expression of OCTN2 and carnitine concentrations in the muscular tissues, liver and kidney [36]. Interestingly, impairment of carnitine absorption in calorie-restricted rats probably attenuates the carnitine concentration increment induced by repeated dietary L-carnitine administration during calorie restriction. Thus, repeated intravenous L-carnitine supplementation may produce a greater increase the total carnitine concentration in skeletal muscle than oral L-carnitine during the modified fasting period.

Likewise, decreased carnitine in muscle, liver and kidney has been found to be a common trait of the insulin resistant state, including diet-induced obesity, genetic diabetes and aging [10]. Thus, these findings prompted us to hypothesize that increases in the total content of carnitine in skeletal muscle may lead to beneficial effects on weight loss and insulin sensitivity. We have demonstrated that intravenous L-carnitine administration enhanced weight loss and insulin concentration reduction during modified fasting therapy. To determine whether increased weight reduction is associated with skeletal muscular carnitine concentration remains to be studied in the future.

Moreover, sitting blood pressure of participants engaging in the modified fasting regimen in the CT group revealed significant reductions, which could be ameliorated by L-carnitine. It has been mentioned that postural hypotension often occurred by the second week of fasting due to dehydration [37]. So we suggested that L-carnitine appears to be able to maintain normal sitting blood pressure by increasing resistance to dehydration during this period.

Lipid profile abnormalities induced by zero-calorie fasting was another controversial issue restricting the application of fasting therapy. As we introduced a 200 Kcal diet to modified fasting, cholesterol abnormalities including increased serum TC, LDL cholesterol and apoB concentrations were found in accordance with zero-calorie fasting [38]. Interestingly, LDL cholesterol was markedly decreased in obese patients during the modified fasting period with a nutritional energy intake of 300 kcal per day [39]. However, the decreased HDL cholesterol and apoA1 concentrations induced by modified fasting are contradictory to that reported in previous studies [38, 40] which showed that these parameters increased during the zero-calorie fasting period. The patients recruited in this study were of older as compared to previous studies, which may have contributed to the variable results. A previous study using labeled cholesterol has demonstrated that the circulating cholesterol level was elevated during the zero-calorie fasting period due to the release of the lipid droplets from adipose tissues [40]. Furthermore, decreased LDL cholesterol uptake by the liver could be an alternative mechanism contributing to increased LDL cholesterol concentrations [41]. Interestingly, our findings showed that incremental TC concentrations were significantly reversed with L-carnitine administration.

Nevertheless, triglycerides and FFA concentrations were reduced for lipolysis during the fasting period, which revealed that L-carnitine administration exerted no obvious effect. Furthermore, our data showed that L-carnitine was unable to reduce the lp(a) concentration increment induced by starvation. It has been suggested that it could significantly reduce the lp(a) concentration in diabetic and non-diabetic patients [42, 43]. Nonetheless, this contradicts the study by Rahbar et al. that reported that oral L-carnitine administration could not significantly reduce lp(a) concentration in type IIdiabetic patients [44]. Accordingly, we hypothesized that metabolic cholesterol abnormalities may be associated with free carnitine depletion during the fasting period, which warrants further investigation.

Hepatic enzyme aberrations, including ALT and AST increases, were observed due to oxidative stress during modified fasting. Furthermore, it has been shown that 24–72 hour zero-calorie fasting causes liver mitochondrial oxidative stress in rats by increasing mitochondrial free radical leak and reactive oxygen species (ROS) generation at complex III level in vivo studies [45, 46]. L-carnitine has been shown to protect human hepatocytes against oxidative stress through its ability to scavenge free radicals [47]. We found that L-carnitine administration was able to attenuate AST and ALT increases during modified fasting.

γ-GGT is widely regarded as a biomarker of alcohol consumption and fatty liver. However, it also has been found to be associated with insulin resistance syndrome including obesity, fat distribution, hypertension, dyslipidemia, and cardiovascular disease [48]. Our results showed that hunger-free fasting was able to reduce γ-GGT concentrations to a greater extent than modified fasting. It could be postulated that hunger-free fasting was more effective than modified fasting in fatty liver management, because 10-20% of the dose may enter the liver during the first 2 hours after administration [13].

Hyperuricemia was found during modified fasting in accordance with previous studies during short-term zero-calorie fasting [49, 50]. Interestingly, previous studies suggested that increased UA may be associated with protein decomposition and forging behavior during the fasting period [51]. Our results showed that hunger-free fasting seemed to slow the increment of UA concentration which confirmed the forging hypothesis. More details about forging behavior during fasting need to be elucidated in further studies. In summary, we hypothesized that hunger-free fasting could be beneficial for long-term weight management for contributing to appetite control.

Despite the limited number of patients, this study revealed no adverse effects with L-carnitine administration. The limited population sample in this study and failure to determine the lean body mass and fat loss in both groups were the two main limitations of this study. Moreover, the intense sensation of hunger in the CT group may force participants to ingest more than allowed by the study design. Nevertheless, the results of this study showed that L-carnitine had a positive impact on weight loss with reduced hunger and improved fatigue.

## Conclusion

In summary, to the best knowledge, the present study is the first to demonstrated that intravenous L-carnitine administration boosted the effects of modified fasting therapy on weight loss and alleviated metabolic abnormalities. L-carnitine infusion also relieved the sensation of hunger and fatigue during the modified fasting period. Our regimen might offer a potential new approach for abdominal obesity management in MetS patients.
